# Parasympathetic Nervous System Functioning Moderates the Associations between Callous-Unemotional Traits and Emotion Understanding Difficulties in Late Childhood

**DOI:** 10.3390/children11020184

**Published:** 2024-02-02

**Authors:** Sarah F. Lynch, Samantha Perlstein, Cora Ordway, Callie Jones, Hanna Lembcke, Rebecca Waller, Nicholas J. Wagner

**Affiliations:** 1 Developmental Sciences, Department of Psychological and Brain Sciences, Boston University, Boston, MA 02215, USA; lynchsf@bu.edu (S.F.L.); cordway@bu.edu (C.O.); 2 Clinical Psychology, Department of Psychology, University of Pennsylvania, Philadelphia, PA 19104, USA; sperl@sas.upenn.edu (S.P.); caljones@sas.upenn.edu (C.J.); 3 Department of General Psychology, University of Hagen, 58097 Hagen, Germany; hannalembcke@gmail.com

**Keywords:** emotion understanding, callous unemotional traits, parasympathetic nervous system, physiological regulation

## Abstract

Background: Callous-unemotional (CU) traits are characterized by low empathy, guilt, and prosociality, putting children at risk for lifespan antisocial behavior. Elevated CU traits have been linked separately to difficulties with emotion understanding (i.e., identifying emotional states of others) and disrupted parasympathetic nervous system (PNS) functioning. However, no study has investigated how PNS functioning and emotion understanding are jointly related to CU traits. Method: We explored associations between CU traits, emotion understanding, and PNS functioning (indexed via respiratory sinus arrhythmia [RSA]) among children aged 7–10 years old (*n* = 55). We also tested whether deficits in emotion understanding differ across specific emotions (i.e., fear, pain, happiness, anger). Each child’s RSA was continuously recorded while they watched a film that included emotionally evocative social interactions. To assess emotion understanding, children identified emotions replayed in 1s animations of scenes from the film. Parents reported on child CU traits, conduct problems, and demographic information. Results: Higher CU traits were related to lower emotion understanding (*β* = −0.43, *p* = 0.03). PNS activity during the film moderated this association (*β* = −0.47, *p* < 0.001), such that CU traits were associated with lower emotion understanding among children with mean (B = −0.01, *t* = −2.46, *p* = 0.02) or high (i.e., 1 *SD* > *M*; B = −0.02, *t* = −3.00, *p* < 0.001) RSA levels during the film, but not among children with low RSA levels (i.e., 1 *SD* < M; B = 0.00, *t* = −0.53, *p* = 0.60). Moreover, we found that the observed moderated associations are driven by deficits in fear, specifically. Conclusions: The link between poorer emotion understanding, fear understanding in particular, and CU traits was attenuated for children who demonstrated patterns of PNS functioning consistent with attentional engagement while viewing the emotion stimuli.

## 1. Introduction

Emotion understanding is the ability to recognize others’ emotions and understand the context within which those emotions are expressed [1,2]. Emotion understanding supports social competence and motivates prosocial behavior [3]. Callous-unemotional (CU) traits are characterized by a lack of empathy, low guilt, and limited prosociality [4,5] and predict risk for severe and chronic antisocial behavior and violence across the lifespan [4]. CU traits have consistently been linked to difficulties recognizing, interpreting, and responding appropriately to the social and emotional cues of others, as evidenced by reduced attention to others’ emotions [6,7], inaccurate labeling of emotions and identification of the causes of emotions expressed in spoken stories [8], and emotion recognition difficulties in the contexts of facial expressions, illustrations, and stories [3]. Prior research has established that many emotion deficits related to CU traits, such as emotion recognition and emotion regulation, occur within the context of negative or ambiguous emotions, such as differentiating between fear, anger, and neutral expressions [9,10,11,12], while other work proposes deficits may be seen across a broader range of emotions [13].

Together, these research findings support a link between emotion understanding difficulties and CU traits. However, prior studies examining emotion understanding are limited by methods that have largely relied on static images or truncated single emotion-specific vignettes [14], which do not require children to incorporate social and contextual information and may not adequately capture the dynamic and complex nature of emotion understanding. Indeed, varying measurement approaches may have contributed to the mixed findings in the literature, with only a handful of prior studies having used narrative-driven, video-based tasks to examine the relations between CU traits and emotion understanding [14]. For example, no link was found between emotion understanding and CU traits in a study of 3 year olds that used a puppet vignette task [15]. In contrast, in a study of 4 to 8 year olds, children with higher CU traits showed lower emotion understanding, operationalized via tasks assessing emotion perception, emotion causes, and ambivalent emotion recognition [16]. To better clarify the links between emotion understanding difficulties and CU traits, studies are needed that examine their associations using video-based tasks that present emotion information in the context of a dynamic narrative. 

In addition, very few studies have explored physiological patterns of functioning that support children’s emotion understanding, which could provide further insight into the potential mechanism underlying the links between CU traits and emotion understanding. The activity of the parasympathetic nervous system (PNS) regulates cardiac output to support adaptive engagement with the environment by regulating the distribution of behavioral, emotional, and attentional resources to maintain homeostasis and meet environmental demands [17]. Respiratory sinus arrhythmia (RSA) is a measure of the inhibitory influence of the PNS, which facilitates metabolic adjustments to respond to the ever-changing environment [18,19] and provides insight into individual differences in the capacity to respond appropriately to environmental challenges [20,21]. 

Adaptive patterns of RSA regulation support effective social interactions, including emotional and social processing (see [22]). For example, in the context of an emotion-related task, a lower task-related RSA relative to baseline or resting RSA can be interpreted as facilitating engagement with salient emotion cues of others, such as pain, anger, and fear [22]. That is, an adaptive diversion of physiological resources (i.e., comparatively lower RSA) from the maintenance of homeostasis in response to emotion cues may allow for engagement with the cues via attending and orienting [19,23,24]. In contrast, a comparatively higher RSA may indicate a disregard for or a failure to engage with or attend appropriately to emotionally challenging stimuli [22,25,26]. 

For example, a lower RSA in response to cues of emotion has been linked to fewer externalizing problems [27] and more adaptive social behavior [19,28,29], whereas higher levels of RSA are thought to underpin many emotion-related difficulties associated with CU traits [22,30]. Most prior studies have examined RSA levels at the baseline (i.e., not in response to emotion or social cues), finding that CU traits are related to a low baseline RSA, suggesting reduced regulatory resources [25] in infancy [31], childhood [32,33], and adolescence [34,35,36]. Among studies that have examined PNS functioning during emotion-related tasks, lower prosociality (a key feature of CU traits) was associated with higher RSA in response to emotional film clips among 7- to 11-year-old children [37]. Wagner and colleagues (2017) report that CU traits in early childhood predict elevated externalizing problems, but only for children demonstrating a higher baseline and little to no RSA suppression in response to a fear stimulus [31]. CU traits were also linked to stable high RSA levels during parent–child social interactions in 10 to 14 year olds [38]. Finally, CU traits were associated with high RSA levels during a virtual reality fear-induction task in 12- to 14-year-old children [39]. In sum, there is preliminary evidence that CU traits are associated with PNS regulation patterns suggestive of inadequate allocation of physiological resources in response to salient emotional or social cues in the environment. However, studies have yet to explore these associations using narrative-driven, video-based tasks designed to induce emotions, and no studies have tested whether PNS functioning moderates the associations between emotion understanding and CU traits in this context. 

The current study aimed to address these gaps in the literature using a narrative-driven animated film depicting complex interactions between characters across a range of emotions. Under our first aim, we tested whether CU traits were related to general emotion understanding deficits. We hypothesized that higher CU traits would be associated with lower emotion understanding accuracy. Second, we tested whether associations between CU traits and emotion understanding varied as a function of PNS functioning during the film, indexed via RSA. We hypothesized that children with higher CU traits and who showed relatively higher RSA throughout the animated film would demonstrate poorer emotion understanding. Third, we aimed to test whether and how the hypothesized emotion understanding deficits, and the moderating role of RSA, varied as a function of emotion type. To address this aim, we first examined the main effect and moderated associations between CU traits, RSA, and deficits in fear, pain, happiness, and anger. Given the extant literature suggesting links between CU traits and deficits in understanding negative emotions specifically, we hypothesized that children would show more deficits in understanding of fear. 

## 2. Methods

### Participants

Participants were recruited from two large northeastern US cities using flyers, online advertisements, social media, and institutionally maintained databases of families who had agreed to be contacted about research participation. Inclusion criteria were (1) child aged 7 to 10 years old, (2) fluency in English, (3) normal or corrected vision, and (4) the participating parent is the primary caregiver who lived with the child ≥50% of the time. Exclusion criteria were (1) diagnosed cognitive or psychiatric disability other than ADHD, (2) developmental delays, and (3) receiving treatment or medication for any psychiatric condition or behavioral issue. Parents provided informed written consent and children provided verbal assent. Families were compensated $120. All procedures were approved by the Institutional Review Boards at Boston University and University of Pennsylvania. The final sample (*n* = 55; Boston University, *n* = 25; University of Pennsylvania, *n* = 30) was diverse in socioeconomic position based on parental education (7.4% no high school degree, 33.4% high school degree, 27.8% bachelor or associate degree, 27.8% graduate degree) and parent reports of child race (46.3% Black or African American, 24.1% White, 9.3% Asian, 16.7% Other) and gender (56.6% female, 43.4% male). 

## 3. Procedure

Data were collected during a 3 h visit at a research lab at Boston University or [University of Pennsylvania. During the lab visit, tasks were completed in one of four blocks that included a baseline block, during which the child quietly colored for 5 min, two blocks of computer-based tasks, and one block of parent–child interaction tasks. While the child completed computer-based tasks, the parent remained in a separate room and completed a demographic interview and questionnaires. Physiological measures for the child were collected continuously throughout the study. Visits were scheduled at the convenience of the families, with most taking place in the morning or afternoon hours. Snacks and breaks were provided during the visit. 

### 3.1. Measures

#### 3.1.1. Emotion Understanding

To assess emotion understanding, children viewed a short, animated film (“Partly Cloudy”, [5 min 30 s]; PIXAR Studios, 2009) on a computer in the lab. The film depicts interactions between two non-human cartoon characters that vary in emotional content (plot description found online at https://www.pixar.com/partly-cloudy#partly-cloudy-1 accessed on 19 December 2023). Prior research has combined viewing this film with functional magnetic resonance imaging (fMRI) to establish that emotionally evocative events during the film are associated with activation in brain regions associated with emotion processing in adults and children [40]. In the current study, children watched the film and were then asked to identify the emotions of the characters. The short film depicts two characters: Gus, a storm cloud who makes dangerous baby animals, and Peck, the stork who delivers them to their families. The film relies entirely on facial expressions, body language, and context to convey the story, as there is no spoken dialogue between the characters. Gus and Peck engage in multiple emotion-driven interactions, such as anger (e.g., Gus is angry when Peck abandons him), pain (e.g., Peck is in pain when Gus hands him a porcupine), happiness (e.g., Gus is happy when Peck returns), and fear (e.g., Peck is afraid of the baby animal Gus has created). Grasping the emotions displayed by each character individually requires an understanding of the context and the intentions of their social partner. 

**Figure 1:**
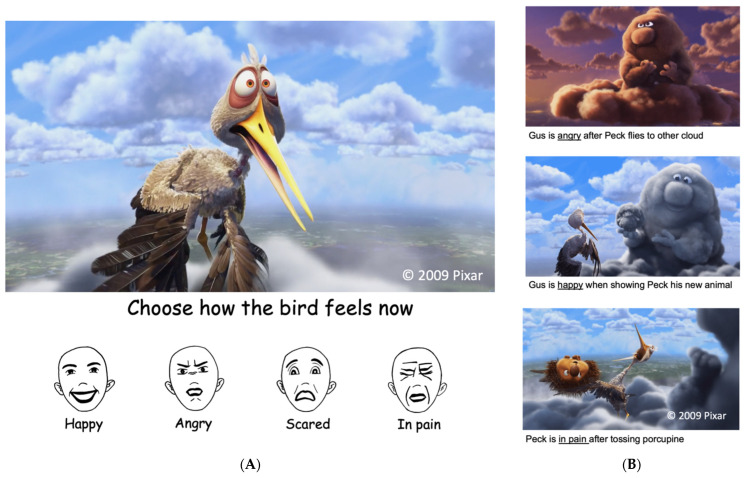
An overview of the Pixar task to assess emotion understanding, including pictorial response scale and stills from GIFs. (**A**) Example presentation of the task screen depicting a still from the GIF of the bird displaying “fear”. Cartoon images of prototypical facial expressions are presented below the film GIFs during the emotion understanding component of the task. GIFs presented in 1 s loops at 38 frames per second. (**B**) Examples of stills depicting angry, happy, and pained emotions during the task. Two different examples of each emotion were presented in separate trials throughout the task (*n* = 10 trials). Images ©2009 Pixar.

Immediately following the film, children were shown a series of Graphics Interchange Format (GIF) images presented individually in 1 s loops at 38 frames per second to scaffold this process. These GIFs depicted happy, fearful, angry, and pained emotions expressed by Gus and Peck during the film (Figure 1). Children were shown a total of 10 GIFs, including 2 GIFs of each emotion. The question, “What was the [character] feeling?” was displayed on the screen for each trial and simultaneously read aloud by the experimenter. GIFs were shown in the same order across participants and displayed alongside a pictorial response scale depicting prototypical facial configurations presented in the same order with the corresponding emotion word displayed below each image [11,41]. Prior to the task, children were asked to identify the emotions depicted in the pictorial response scale to ensure they recognized each basic emotion representation. During the emotion selection portion of the task, children used the mouse to click on the response option that they thought corresponded to the emotion being shown by the characters in each GIF. The proportion of emotions identified correctly in this task provided an index of emotion understanding. Emotion-specific understanding was also calculated for each emotion separately. 

#### 3.1.2. RSA 

Heart rate was assessed via electrocardiogram (ECG) signals, recorded continuously with a BIOPAC data acquisition system (MP160 Windows), using an electrocardiogram amplifier (ECG100C) and AcqKnowledge software (Version 4.3.1. BIOPAC Systems Inc., Goleta, CA, USA). Recording electrodes were placed at the top center of the chest (10 cm below the suprasternal notch) and at the bottom left and right of the ribs (10 cm above the bottom of the rib cage). The ECG signal was further processed by manually inspecting the detected R peaks and valid interbeat intervals (IBI), which represent the time elapsed between consecutive heartbeats, using the CardioEdit Plus v.1 software (Brain-Body Center, University of Illinois at Chicago, Chicago, IL, USA). IBI data were visually inspected and edited offline via CardioEdit by excluding outliers or by making arithmetic corrections to the intervals [42]. RSA values were derived from IBI data defined as the variance within the age-specific respiration frequency band associated with spontaneous breathing using CardioBatch software (Brain-Body Center, University of Illinois at Chicago). The amplitude of RSA values was calculated as the natural logarithm of the variance across the duration of the task, consistent with the procedures developed by [43]. 

An average of RSA across the film clip was used to index individual variability in PNS functioning for each child. RSA during the baseline task was included as a covariate in all analyses to isolate the associations between levels of RSA during the film and study outcomes. Simulation studies have demonstrated that adjusting for autoregressive effects (e.g., examining RSA during the film, controlling for RSA during the baseline task) provides superior statistical estimation as compared to change scores or percent change from baseline scores [44]. Comparatively lower levels of RSA during the film clip suggest PNS withdrawal in response to the stimuli. Studies examining RSA functioning across a specific context have adopted similar autoregressive approaches (see [45,46,47]). 

#### 3.1.3. CU Traits 

CU traits were measured using parent reports on the Inventory of Callous Unemotional Traits (ICU; [48]), a 24-item measure that assesses callousness, uncaring, and unemotionality, with items rated on a four-point scale (0 = not at all true; 3 = definitely true). The ICU is an assessment tool designed to measure traits associated with callousness and lack of empathy in children. It includes items related to the child’s interpersonal style, emotional expression, and behavioral tendencies. The ICU has been validated in clinical and community samples of children and adolescents [49,50]. The internal consistency was high (α = 0.86).

#### 3.1.4. Covariates 

Conduct Problems (CP) were assessed using the 5-item Conduct Problem subscale of the parent-reported Strengths and Difficulties Questionnaire (SDQ; [51]), with parents rating their child’s behavior in the last six months (e.g., often loses temper). Using a three-point scale (0 = not true; 2 = certainly true). As CU traits are often co-occurring with CP, including CP as a covariate isolates the effects of CU traits specifically. The CP subscale covers a range of conduct-related behaviors, including aggression, defiance, and rule-breaking. The internal consistency of the CP subscale was acceptable (α = 0.75). Child age, gender, parent education, and race were reported by parents during a demographic interview. The study site was also included as a covariate in all models. 

### 3.2. Analytic Plan

Study hypotheses were tested by estimating saturated path models in Mplus 8.3 [52] using full information maximum likelihood (FIML) [53]. The first set of models examined the links between CU traits and overall emotion understanding performance. In these models, we first tested whether CU traits and task RSA were independently related to emotion understanding, controlling for baseline RSA, CP, and covariates. Second, we created a product term of mean-centered CU traits and task RSA scores (i.e., interaction) to test whether the relationship between CU traits and emotion understanding varied as a function of task RSA. We probed interactions using standard recommendations, including exploring associations between CU traits and emotion understanding at one *SD* above and below the mean for RSA. Second, we used regions of significance (RoS) analyses to identify the range of values of RSA for which CU traits predicted emotion understanding [54]. 

The next set of models were designed to test the potential links between CU traits, RSA, and emotion-specific deficits (e.g., happiness, fear, anger, pain). A series of saturated path models were estimated to (1) test whether CU traits predicted performance on each specific emotion, controlling for performance on the other emotion categories and other covariates, and (2) examine whether the relationship between CU traits and specific emotion performance varied as a function of task RSA. The final interaction model was informed by preceding models and included fear and pain as covarying outcomes to directly test their relative associations with CU traits and task RSA. Similar to the first set of models, significant interactions were probed following established procedures. 

## 4. Results

**Figure 2:**
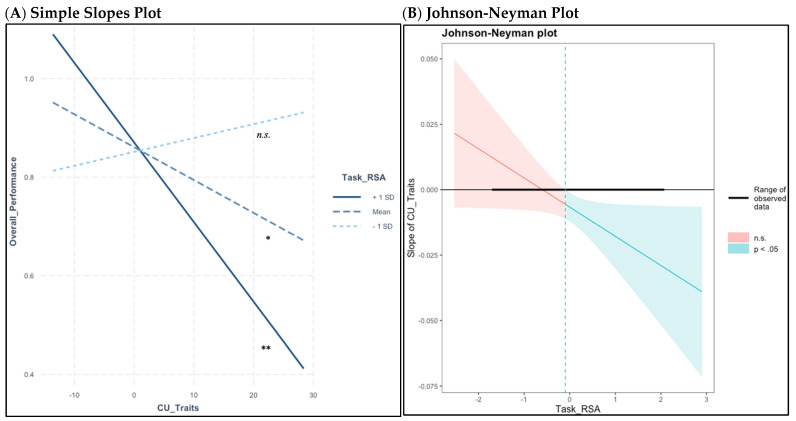
Higher levels of CU traits are related to lower emotion understanding accuracy at mean and high levels of RSA during the animated film, but not at low levels of RSA. Note: * *p* < 0.05, ** *p* < 0.01, *n.s.* = Not Significant. (**A**) Simple slopes plot showing that higher CU traits were associated with lower emotion understanding accuracy for children showing mean (B = −0.01, *t* = −2.46, *p* = 0.02) or high levels (i.e., 1 *SD* > *M*; B = −0.02, *t* = −3.00, *p* < 0.001) of RSA during the animated film, but not for children with low levels of RSA (i.e., 1 *SD* < *M*; B = 0.00, *t* = −0.53, *p* = 0.60). (**B**) Johnson-Neyman plot showing the range of significant slope values for the prediction of emotion understanding accuracy using CU traits for children just below mean task RSA and above (upper bound threshold of −0.10, range of observed, centered values were −1.68 to 2.05). The lower bound RoS for RSA was outside the data range and was not interpreted.

Table 1 presents descriptive statistics and bivariate correlations for study variables. First, a saturated path model testing main effects showed that CU traits were related to lower emotion understanding accuracy (B = −0.01, *β* = −0.43, *p* = 0.03) (Table 2, Model 1). The interaction model showed that this relationship was moderated by task RSA (B = −0.02, *β* = −0.45, *p* < 0.001) (Table 2, Model 2). Simple slopes analyses demonstrated that higher CU traits were associated with lower emotion understanding accuracy for children with mean (B = −0.01, *t* = −2.46, *p* = 0.02) or higher levels (i.e., 1 *SD* > *M*; B = −0.02, *t =* −3.00, *p* < 0.001) of task RSA, but not for children with low task RSA (i.e., 1 *SD* < *M*; B = 0.00, *t* = −0.53, *p* = 0.60) (Figure 2A). The RoS analysis established that higher CU traits were related to lower emotion understanding accuracy when children were just below or above the mean for RSA during the film (upper bound threshold of −0.10, range of observed, centered values were −1.68 to 2.05; Figure 2B). The lower bound RoS for RSA was outside the data range and was not interpreted. 

**Figure 3:**
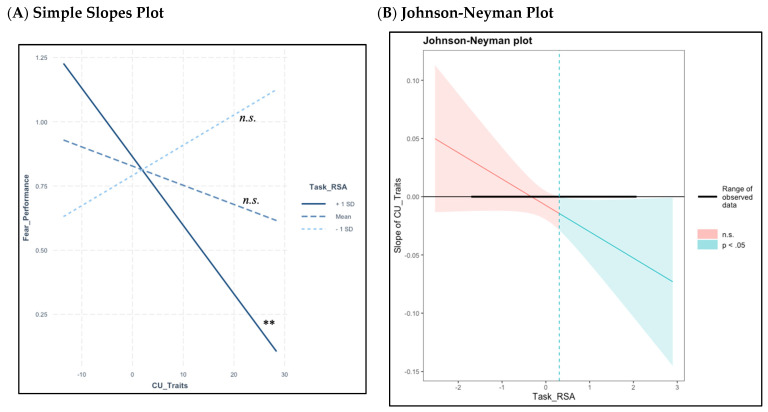
Higher levels of CU traits are related to lower fear accuracy at mean and high levels of RSA during the animated film, but not at low levels of RSA. Note: * *p* < 0.05, ** *p* < 0.01, *n.s.* = Not Significant. (**A**) Simple slopes plot showing that higher CU traits were associated with lower emotion understanding accuracy for children showing high levels (i.e., 1 *SD* > *M*; B = −0.03, *t* = −2.23, *p* = 0.03) of RSA during the animated film, but not for children with mean (B = 0.01, *t* = −0.99, *p* = 0.33) or low levels (i.e., 1 *SD* < *M*; B = −0.01, *t* = −1.24, *p* = 0.22) of RSA. (**B**) Johnson-Neyman plot showing the range of significant slope values for the prediction of emotion understanding accuracy using CU traits for children just below mean task RSA and above (upper bound threshold of −0.10, range of observed, centered values were −1.68 to 2.05). The lower bound RoS for RSA was outside the data range and was not interpreted.

Table 3 presents a set of saturated path models testing the main effects of CU traits and RSA on the prediction of each emotion (pain, fear, anger, happiness), controlling for the other emotions and covariates. Results suggest that CU traits only directly predict emotion understanding accuracy for pain (B = −0.01, *β* = −0.36, *p* = 0.02). Individual interaction models (Table 4) showed that the interaction between CU traits and task RSA significantly predicted both pain (B = −0.02, *β* = −0.37, *p* = 0.01) and fear (B = −0.04, *β* = −0.60, *p* < 0.001). when controlling for the other emotions. To further clarify these associations, pain and fear performance were included as covarying outcomes and regressed on model predictors (Table 5). Results suggest that the interaction between CU traits and task RSA predicts fear (B = −0.04, *β* = −0.37, *p* < 0.0001) but not pain when predicted simultaneously, accounting for their overlap via the inclusion of their covariance. Happiness and anger were retained as covariates in this model. Simple slopes analyses revealed that higher CU traits were associated with lower emotion understanding accuracy of fear for children with higher levels (i.e., 1 *SD* > *M*; B = −0.03, *t* = −2.23, *p* = 0.03) of task RSA, but not for children with mean (B = 0.01, *t* = −0.99, *p* = 0.33) or low (i.e., 1 *SD* < *M*; B = −0.01, *t* = −1.24, *p* = 0.22) task RSA (Figure 3A). The RoS analysis established that higher CU traits were related to lower emotion understanding accuracy of fear when children were inside the mean interval for RSA during the film (0.31, 3.40; range of observed, centered values were −1.68 to 2.05; Figure 3B). 

## 5. Discussion

Emotion understanding supports healthy socioemotional development in children, whereas emotion understanding difficulties are associated with many forms of psychopathology, including the presence of CU traits. Consistent with prior research, higher CU traits were associated with lower emotion understanding accuracy overall [3,6,7,8]. The current findings also suggest that these effects may be driven by deficits in fear accuracy. Prior studies assessing emotion understanding have used tasks that assess knowledge and recognition of emotions, but only a few have used narrative-driven video measures (see [14] for review). The current study advances the literature by using an animated film that required children to interpret and select the emotions of multiple characters within a narrative context rather than solely relying on static features of an image. Our findings are generally consistent with previous studies that assessed emotion understanding using narrative-based tasks [16,55].

In addition, we provide insight into the neurophysiological mechanisms underpinning associations between emotion understanding difficulties and CU traits. There was no association between CU traits and emotion understanding for individuals demonstrating comparatively lower levels of RSA during the film. Within trials specific to each emotion, only the accuracy of understanding pain had direct associations with CU traits. However, the interaction between CU traits and task RSA predicted the accuracy of understanding fear but not pain when accounting for their shared variance. Specifically, results suggest that higher CU traits were associated with lower emotion understanding accuracy, and lower fear accuracy in particular, but only among children displaying comparatively higher RSAs during the film. Taken together, results are consistent with the literature suggesting that the PNS may support emotion understanding by facilitating engagement with emotion stimuli, and that activation of the PNS (i.e., comparatively lower task RSA as compared to baseline RSA) attenuates links between deficits in emotion understanding and CU traits. 

Reduced activation of the PNS while viewing social and emotional stimuli was associated with lower emotion understanding and may therefore represent one mechanism underlying established links between emotion-related difficulties and CU traits. Our findings suggest important heterogeneity in patterns of PNS functioning while engaging with emotional content that could provide insight into the etiology of CU traits in childhood and potential treatment targets, including interventions that directly target physiological regulation [56,57]. For example, meta-analytic evidence suggests that variability biofeedback improves a range of symptoms and functioning and may represent a useful complementary treatment [58], while Autonomic Nervous System Biofeedback Modality treatment has shown some efficacy in the treatment of attention problems [59]. 

Additionally, our findings highlight the need for future research examining links between emotion deficits and CU traits to consider the nuances across various emotion-specific stimuli. Our results are consistent with other research suggesting that CU traits may be associated with deficits in recognizing negative emotions specifically [31,60], and that PNS functioning may support emotion recognition and regulation in the context of negative emotions, including fear [37,47,61,62]. However, more work should be performed to elucidate the attentional and regulatory mechanisms underlying these deficits and whether and how children’s attention and physiological regulation might further our understanding of variation in performance among negative emotion categories (e.g., pain, fear). 

In addition to research linking CU traits with poorer understanding and recognition of negative emotions, particularly cues of social threat, such as fear, anger, and sadness [9,12,63,64], additional research suggests that these deficits may be driven by a lack of attention to salient emotional features including the eye region [65]. Indeed, children and adolescents with CU traits appear to make less eye contact with caregivers [66,67] and focus less on the eye region during emotion recognition tasks [68]. Studies that incorporate information about specific attentional processes in the context of emotion understanding and recognition are important, but results from the current study, and others exploring links between children’s physiology and emotion, suggest that indices of PNS functioning during emotion experiences may provide additional insight into observed deficits associated with CU traits. Indeed, interventions that promote reciprocal eye gaze have reported mixed results [69,70], highlighting persistent gaps in our understanding of the specific mechanisms underlying the links between CU traits and emotion deficits. 

The current study used a rigorous multi-method approach, including continuous physiological recording during a narrative-based video task used to assess emotion understanding. Nevertheless, the findings should be viewed alongside several key limitations. First, the sample size was small and prohibited examining epoch-by-epoch associations between PNS functioning during the film. Moreover, we tested a community sample, reducing the generalizability of findings to children in clinic or juvenile justice settings. Second, although we took advantage of continuous physiological recording to index engagement with social and emotional content, we did not assess attention. Thus, future research combining physiological and attentional measures (e.g., eye-tracking) would help to clarify whether links between RSA, CU traits, and lower emotion understanding are the consequence of difficulties attending to the salient portions of stimuli or deficits in arousal and engagement despite the adequate allocation of attention [67]. Finally, this was a cross-sectional and observational study, limiting implications about temporal or causal relationships between variables. Future research is needed to clarify how links between PNS functioning, emotion understanding, and the development of CU traits unfold over time and across a variety of contexts, including experimental conditions that manipulate or promote physiological engagement in different ways [71]. 

In sum, CU traits are associated with difficulties in understanding the emotions of others, as evidenced using a narrative-driven video task that required children to appreciate feelings around loyalty, betrayal, and friendship. There was evidence that emotion understanding difficulties were specific to negative emotions including fear and were most strongly associated with CU traits when children showed reduced PNS activation (i.e., comparatively higher levels of RSA) while watching the film. These findings suggest that the PNS should be considered as a potential mechanism underlying treatment response in the context of emotion skills training (e.g., [71]) and that future research should consider whether physiological [71] and attentional [66] processes could be considered treatment targets themselves to facilitate adaptive engagement with social or emotional environmental cues. 

## Figures and Tables

**Table 1:** Zero-order bivariate correlations between study variables.

	Site	Age	Gender	Race	Parent Edu.	Conduct Problems	Baseline RSA	CU Traits	Film RSA	Emot. Understanding
Site	-									
Child age	−0.06	-								
Child gender	−0.01	−0.08	-							
Child race	−0.27	0.15	0.05	-						
Parent education	−0.47 **	−0.17	−0.06	0.38 **	-					
Conduct problems	0.15	−0.16	−0.05	−0.14	0.10	-				
RSA at baseline	0.35 *	0.24	0.10	0.11	−0.11	0.09	-			
CU Traits	0.11	−0.19	0.22	−0.05	0.07	0.60 **	0.04	-		
Avg. RSA during film	0.14	0.35 *	−0.05	−0.11	−0.33 *	−0.01	0.48 **	0.00	-	
Emot. understanding accuracy (% correct)	−0.11	0.31 *	0.20	−0.12	0.05	−0.04	0.13	−0.28	0.14	-
*M*	1.54	9.03	0.43	0.25	3.06	1.14	6.86	15.61	7.24	0.86
*SD*	0.50	1.14	0.50	0.44	1.79	1.69	1.05	8.70	0.85	0.15
Min	1.00	7.08	0.00	0.00	0.00	0.00	4.83	2.00	5.56	0.38
Max	2.00	10.92	1.00	1.00	6.00	9.00	9.56	44.00	9.29	1.00
*n*	55	53	53	52	53	51	45	51	41	51

Note. * *p* < 0.05, ** *p* <0.01.

**Table 2:** Unstandardized and standardized estimates from path models examining direct and interactive associations between CU traits and average RSA during the film and emotion understanding accuracy across all emotions.

	Model 1, Main Effects		Model 2, Interactive Effects	
	*Emotion Understanding Accuracy (% Correct)*			
	B (*β*)	95% Confidence Interval	B (*β*)	95% Confidence Interval
*Main effects*				
Site	−0.04 (−0.13)	−0.11, 0.04	−0.03 (−0.09)	−0.12, 0.06
Child age	0.05 (0.34) **	0.01, 0.08	0.06 (0.47) **	0.04, 0.09
Child gender	0.11 (0.34) **	0.04, 0.17	0.11 (0.37) **	0.04, 0.18
Child race	−0.09 (−0.26) *	−0.18, −0.002	−0.10 (−0.29) *	−0.2, −0.002
Parent education	0.01 (0.16)	−0.01, 0.04	0.01 (0.06)	−0.01, 0.02
Conduct problems	0.03 (0.27) *	0.002, 0.05	0.03 (0.29) **	0.01, 0.05
Baseline RSA	0.02 (0.13)	−0.03, 0.06	0.00 (0.00)	−0.06, 0.06
CU Traits	−0.01 (−0.43) *	−0.02, −0.001	−0.01(−0.47) **	−0.01, −0.004
Avg. RSA during film	0.01 (0.03)	−0.05, 0.06	0.01 (0.03)	−0.05, 0.06
*Interaction*				
CU traits × Avg. RSA during film			−0.02 (−0.45) **	−0.02, −0.01

Note. * *p* < 0.05, ** *p* < 0.01.

**Table 3:** **Main Effects, Emotion Specific.** Unstandardized and standardized estimates from path models examining associations between CU traits and average RSA during the film and emotion understanding accuracy, exploring each emotion as individual outcomes.

	Fear		Pain		Happy		Anger	
	*Emotion Understanding Accuracy (% Correct)*							
	B (*β*)	95% CI	B (*β*)	95% CI	B (*β*)	95% CI	B (*β*)	95% CI
*Main effects*								
Site	−0.06 (−0.10)	−0.23, 0.11	−0.01 (−0.02)	−0.13, 0.11	0.06 (0.11)	−0.16, 0.28	−0.08 (−0.23)	−0.20, 0.04
Child age	0.11 (0.38) *	0.02, 0.19	0.04 (0.19)	−0.02, 0.11	0.02 (0.07)	−0.08, 0.12	0.00 (0.01)	−0.04, 0.04
Child sex	0.21 (0.32) **	0.06, 0.35	0.11 (0.21)	−0.03, 0.25	0.12 (0.21)	−0.09, 0.32	−0.03 (−0.09)	−0.14, 0.07
Child race	−0.04 (−0.06)	−0.26, 0.17	0.01 (0.01)	−0.19, 0.21	−0.17 (−0.26)	−0.41, 0.07	−0.10 (−0.24)	−0.24, 0.04
Parent education	0.02 (0.12)	−0.19, 0.83	0.02 (0.12)	−0.02, 0.06	0.01 (0.08)	−0.13, 0.49	0.00 (−0.04)	0.06, 0.39
Conduct problems	0.02 (0.11)	−0.32, 0.30	−0.01 (−0.08)	−0.06, 0.04	0.05 (0.30)	−0.77, 0.14	0.04 (0.33) *	−0.27, 0.04
Baseline avg. RSA	−0.01 (−0.02)	−0.53, 0.25	0.02 (0.08)	−0.05, 0.10	0.00 (0.00)	−0.29, 0.27	0.03 (0.14)	−0.07, 0.27
CU traits	−0.01 (−0.17)	−0.02, 0.07	−0.01 (−0.36) *	−0.02, 0.00	−0.01 (−0.19)	−0.03, 0.06	0.00 (−0.07)	−0.03, 0.02
Avg. RSA during film	0.07 (0.19)	−0.05, 0.09	0.03 (0.08)	−0.09, 0.14	0.00 (0.01)	0.00, 0.10	−0.06 (−0.28)	0.00, 0.07

Note. * *p* < 0.05, ** *p* <0.01; Note. All emotions included as covariates.

**Table 4:** **Interactions, Emotion Specific.** Unstandardized and standardized estimates from path models examining direct and interactive associations between CU traits and average RSA during the film and emotion understanding accuracy, exploring each emotion as individual outcomes.

	Fear		Pain		Happy		Anger	
	*Emotion Understanding Accuracy (% Correct)*							
	B (*β*)	95% CI	B (*β*)	95% CI	B (*β*)	95% CI	B (*β*)	95% CI
*Main effects*								
Site	−0.02 (−0.04)	−0.19, 0.15	0.00 (−0.01)	−0.14, 0.13	0.07 (0.13)	−0.15, 0.29	−0.08 (−0.23)	−0.20, 0.04
Child age	0.17 (0.61) **	0.08, 0.26	0.09 (0.39) *	0.01, 0.17	0.02 (0.08)	−0.10, 0.14	0.03 (0.17)	−0.03, 0.09
Child sex	0.27 (0.42) **	0.11, 0.42	0.16 (0.31) *	0.01, 0.32	0.12 (0.22)	−0.08, 0.33	0.00 (0.00)	−0.11, 0.11
Child race	−0.09 (−0.12)	−0.31, 0.13	−0.03 (−0.05)	−0.24, 0.18	−0.16 (−0.24)	−0.38, 0.07	−0.12 (−0.28)	−0.25, 0.01
Parent edu.	0.00 (0.01)	−0.04, 0.04	0.01 (0.06)	−0.03, 0.05	0.01 (0.08)	−0.04, 0.06	−0.01 (−0.08)	−0.04, 0.02
Conduct problems	0.03 (0.18)	−0.03, 0.10	0.00 (−0.01)	−0.05, 0.04	0.05 (0.31)	0.00, 0.10	0.04 (0.35) *	0.01, 0.07
Baseline avg. RSA	−0.07 (−0.22)	−0.18, 0.05	−0.01 (−0.05)	−0.09, 0.06	−0.01 (−0.05)	−0.13, 0.11	0.02 (0.10)	−0.04, 0.07
CU traits	−0.01 (−0.27)	−0.02, 0.00	−0.01(−0.43) **	−0.02, 0.00	−0.01 (−0.20)	−0.02, 0.01	0.00 (−0.13)	−0.01, 0.01
Avg. RSA during film	−0.01 (−0.02)	−0.12, 0.10	−0.01 (−0.02)	−0.12, 0.11	0.01 (0.04)	−0.13, 0.15	−0.08 (−0.36) *	−0.14, −0.01
*Interaction*								
CU traits × Avg. RSA during film	−0.04(−0.60) **	−0.06, −0.02	−0.02(−0.37) *	−0.04, −0.01	0.00 (−0.01)	−0.03, 0.03	−0.01 (−0.27)	−0.03, 0.01

Note. * *p* < 0.05, ** *p* < 0.01; Note. All emotions included as covariates.

**Table 5:** Unstandardized and standardized estimates from path models examining direct and interactive associations between CU traits and average RSA during the film and emotion understanding accuracy, with fear and pain included as covarying outcomes.

	Fear		Pain	
	*Emotion Understanding Accuracy (% Correct)*			
	B (*β*)	95% CI	B (*β*)	95% CI
*Main effects*				
Site	−0.02 (−0.04)	−0.19, 0.14	0.00 (0.01)	−0.13, 0.13
Child age	0.15 (0.55) **	0.07, 0.24	0.05 (0.23)	−0.01, 0.12
Child sex	0.24 (0.37) **	0.09, 0.38	0.11 (0.2)	−0.02, 0.23
Child race	−0.09 (−0.12)	−0.30, 0.12	−0.01 (−0.01)	−0.21, 0.19
Happy accuracy	−0.05 (−0.04)	−0.36, 0.26	0.13 (0.14)	−0.07, 0.33
Anger accuracy	−0.03 (−0.02)	−0.46, 0.40	0.33 (0.23)	−0.19, 0.84
Parent education	−0.002 (−0.01)	−0.04, 0.04	0.01 (0.06)	−0.03, 0.05
Conduct problems	0.04 (0.20)	−0.03, 0.11	−0.01 (−0.06)	−0.06, 0.04
Baseline avg. RSA	−0.07 (−0.23)	−0.18, 0.04	0.00 (0.01)	−0.07, 0.07
CU traits	−0.01 (−0.18)	−0.02, 0.00	−0.01 (−0.38) **	−0.02, 0.00
Avg. RSA during film	−0.01 (−0.02)	−0.13, 0.11	−0.01 (−0.02)	−0.13, 0.12
*Interaction*				
CU traits × avg. RSA during film	−0.04 (−0.55) **	−0.05, −0.02	−0.01 (−0.22)	−0.03, 0.00

Note. * *p* < 0.05, ** *p* < 0.01.

## Data Availability

Data will be made available upon reasonable request.
